# Integrating morphological and molecular characterization to reveal genetic diversity and breeding potential in Turkish local alfalfa (*Medicago sativa* L.) populations

**DOI:** 10.1038/s41598-026-52782-3

**Published:** 2026-05-28

**Authors:** Sebahattin Albayrak, Mevlüt Türk

**Affiliations:** 1 Department of Field Crops, Faculty of Agriculture, Ondokuz Mayıs University, Samsun, Turkey; 2 Department of Field Crops, Faculty of Agriculture, Isparta University of Applied Sciences, Isparta, Turkey

**Keywords:** *Medicago sativa* L., Genetic diversity, ISSR markers, PCA-biplot, Phenotypic variation, Forage quality, Lake Region, Ecology, Ecology, Genetics, Plant sciences

## Abstract

Alfalfa (Medicago sativa L.) is one of the most important forage crops due to its high crude protein content, nitrogen fixation ability, and adaptability to diverse ecological conditions. This study aimed to evaluate the morphological, phenological, yield, quality, and molecular diversity of 60 local alfalfa populations, together with 4 control varieties (64 genotypes in total), collected as clones from the Lakes Region of Türkiye (Isparta, Burdur, Afyonkarahisar, and Konya) between 2011 and 2013. Twenty-seven agronomic, morphological, and quality traits were analyzed, revealing substantial phenotypic variation among genotypes in terms of growth habit, regrowth rate, dormancy level, forage yield, and quality. Heatmap, PCA-biplot, and correlation analyses identified five major morphological groups with distinct yield–quality relationships. Molecular clustering and PCoA analyses supported these groupings and revealed strong genetic differentiation shaped by eco-geographic isolation and local adaptation. The combined evaluation of morphological and molecular data demonstrated that several genotypes possess both high agronomic performance and distinct genetic structure. In particular, genotypes LC3, LC17, LC24, LC29, LC39, LC49, LC53, LC54, LC57, and LC60 were identified as the most promising candidates for synthetic variety development due to their superior yield, quality traits, and representation of different genetic clusters. Overall, the findings highlight the significant breeding potential of local alfalfa germplasm and provide a reliable framework for developing high-yielding and high-quality varieties adapted to regional conditions.

**Supplementary Information:**

The online version contains supplementary material available at 10.1038/s41598-026-52782-3.

## Introduction

Alfalfa (*Medicago sativa* L.) is one of the most widely cultivated perennial legume forage crops worldwide^1^. Alfalfa is described as both the king^2^ and queen^3^ of forage crops. Alfalfa is of strategic importance both ecologically and economically due to its high crude protein content, biological nitrogen fixation, and capacity to increase soil fertility^4,5^. Alfalfa^6^, which has a wide ecological distribution due to its ability to adapt to drought, low temperatures, and different soil conditions, is one of the key species determining feed quality in animal production^7,8^. It is also considered an important component of sustainable agricultural systems due to its carbon sequestration, contribution to the nitrogen cycle, and role in erosion control^9^.

Morphological and phenological characteristics are among the most important factors determining alfalfa’s environmental adaptation and productivity^10,11^. Characteristics such as dormancy level^12^, plant coverage area^13^, growth rate, and regrowth ability^14^ reflect the responses of genotypes to environmental stresses and their regional adaptation abilities^15^. The quantitative nature of these traits is strongly influenced by both genetic and environmental effects, resulting in significant variation among populations in different ecological regions^16,17^. Genotypic assessments conducted in regions with climatic and topographic differences bear the traces of both natural selection and local adaptation^18^.

Forage yield and quality constitute the main objectives of alfalfa breeding programs^19^. Dry matter yield, protein content, acid detergent fiber (ADF) and neutral detergent fiber (NDF) ratios are decisive parameters in terms of both animal performance and forage digestibility^20,21^. The complex relationships between these traits highlight the need to minimize quality losses while aiming for yield increases^22^. In this context, the use of multiple statistical analyses (e.g., PCA, correlation matrix) increases the effectiveness of selection strategies by revealing the structural relationships between traits^23^.

Molecular characterization studies are a powerful tool for understanding genetic variation and phylogenetic relationships among plant populations. ISSR (Inter Simple Sequence Repeat) markers are widely used due to their ability to detect high levels of genetic polymorphism, as well as their simplicity, cost-effectiveness, and low DNA requirement^24–26^. These features make ISSR markers a reliable approach for assessing genetic diversity and supporting breeding programs^27^.

Progress in increasing alfalfa biomass yield has stagnated, with only a 1% annual genetic gain reported over the last 20 years^28^. To improve this situation, new breeding strategies need to be developed^29^. Alfalfa exhibits a wide adaptability to different environments^30^. A breeder must recommend suitable alfalfa varieties for different environments; selection in a breeding program is primarily based on yield stability and high quality^31^.

Heat maps, PCA, PCoA, and correlation analyses were performed using 27 morphological and quality parameters, and the genetic diversity of the populations was determined using ISSR markers. The main objective of the study was to determine the phenotypic and genetic diversity levels of genotypes collected from different ecological conditions, analyze this diversity, and propose genotypes with high agronomic performance and genetic distinctiveness as candidates for synthetic variety development in breeding programs.

## Materials and methods

### Plant material and study area

This study was conducted on 60 different alfalfa (Medicago sativa L.) genotypes, together with 4 control varieties (64 genotypes in total), collected as clones from 22 districts in the Lakes Region of Türkiye (Isparta, Burdur, Afyonkarahisar, and Konya) between 2011 and 2013. Although the plant materials were collected between 2011 and 2013, they represent stable genetic resources and are still relevant to current breeding programmes. The collection of plant materials used in this study was conducted in accordance with national regulations in Türkiye, and permission for sample collection was obtained through institutional authorization from Isparta University of Applied Sciences, Faculty of Agriculture. Although the plant materials were originally collected as populations from different locations, individual plants were clonally propagated and evaluated separately; therefore, each experimental unit represents a genotype derived from a population.

### Fieldwork and plant collection

**Fig. 1:**
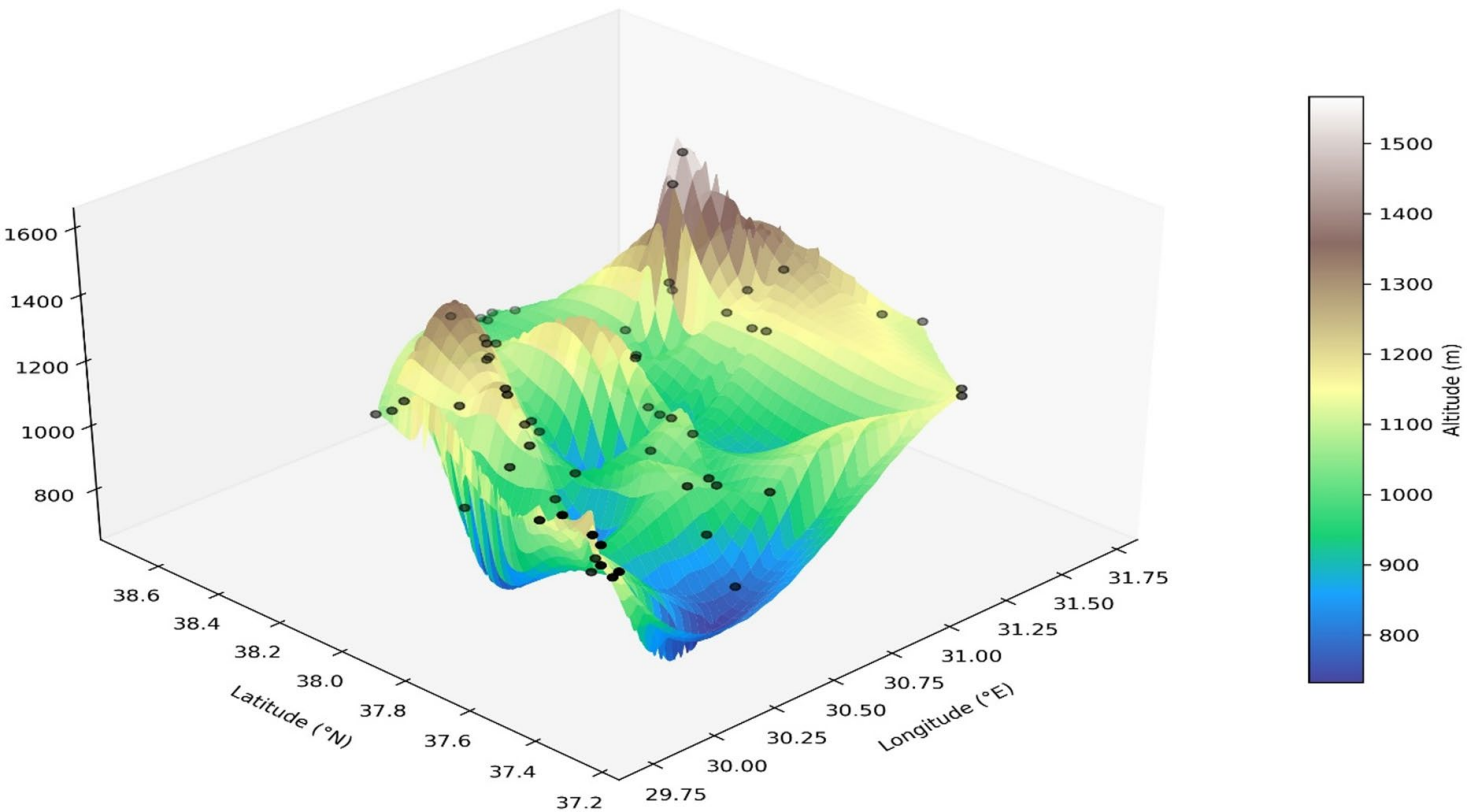
Three-dimensional topographic relief map of the Lakes Region (Türkiye) showing the spatial distribution of 60 wild *Medicago sativa L.* populations. LC codes indicate sampling locations, with altitude gradients represented by terrain color transitions.

Alfalfa materials were collected from 22 districts, starting on 12 May 2011, with stops at 3–5 km intervals^32^. The coordinates and elevation values of the sampling locations above sea level were recorded using a GPS device (Fig. 1).

Three to five plants with roots were taken from each population, planted in 25 × 30 cm pots, and brought to the province of Isparta (37.8102 N, 30.5565 E; 1100 m). To obtain new plants from clones with rooting or survival problems, the budded shoot tips were dipped in a 500 ppm IBA solution for 4–5 s and planted in a peat + perlite (1:1) mixture^33^. After the clones reached sufficient root length within 15–25 days, they were transferred to bags containing a mixture of peat + perlite + sand and left to grow in the greenhouse and open field until September 28, 2011.

**Fig. 2:**
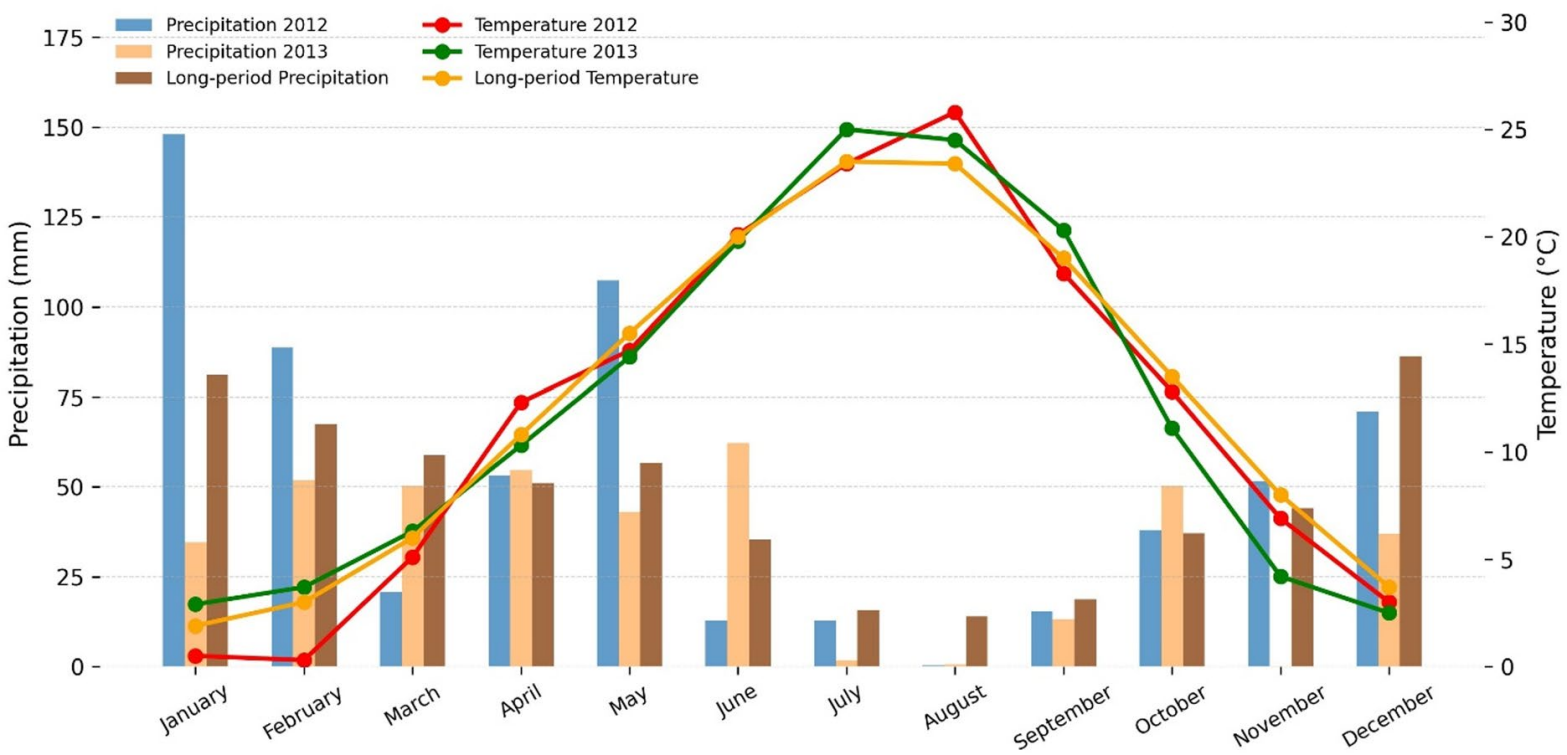
Precipitation and temperature values ​​for the trial years 2012, 2013 and Long-periods.

A total of 2.400 plants (40 plants × 60 genotypes) were transplanted into the field at 80 × 80 cm intervals. Each genotype was represented by 40 plants within the experimental area. The soil in the trial area is clayey, with a pH of 7.05 (neutral) and 1.08% organic matter content. Phosphorus levels were found to be high (45 ppm), while potassium levels were low (67.2 ppm). Rainfall amounts for the trial years were 620 mm in 2012 and 400 mm in 2013, with 2012 exceeding the long-term average (566.4 mm). Temperatures were also slightly below the long-term average (Fig. 2).

### Phenological and morphological observations

Phenological, morphological, and yield-related measurements and observations were made on genotypes planted in the field. For clarity, the evaluated traits were grouped into morphological (e.g., plant height, leaf size, and root crown characteristics), phenological (e.g., flowering time and developmental stages), and yield and quality traits throughout the study. These observations were conducted according to the standard characterization criteria established by the USDA–ARS National Genetic Resources Program and Prosperi et al.^34^. The main characteristics evaluated are presented in Table 1. Crude protein, acid detergent fiber (ADF), and neutral detergent fiber (NDF) analyses in alfalfa genotypes were initially performed according to the methods reported by Albayrak and Türk^35^.

**Table 1:** In alfalfa population’s traits studied.

Number	Parameters
PR1	Winter dormancy (1-dormant; 9-non-dormant)
PR2	Spring plant coverage (1-narrow, 9-wide)
PR3	Summer plant coverage (1-narrow, 9-wide)
PR4	Autumn plant coverage (1-narrow, 9-wide)
PR5	Winter plant coverage (1-narrow, 9-wide)
PR6	Growth rate after mowing (2 weeks after mowing; 1-very slow, 9-very fast)
PR7	Plant resistance after the second year (% number of surviving plants)
PR8	Spread (% soil surface coverage)
PR9	Weed infestation (1-low, 9-high)
PR10	Growth form (1-upright, 9-lying)
PR11	Natural plant height (cm)
PR12	Main stem length (cm)
PR13	Leaf size (1-small; 9-very large)
PR14	Root crown diameter (1-narrow, 5-wide)
PR15	Number of branches on the root crown (number)
PR16	Number of buds on the root crown (number)
PR17	Root crown depth (1 at the surface, 5 at the depth)
PR18	Harvested area per plant (cm²)
PR19	Number of days until flowering begins
PR20	Number of days until flowering ends
PR21	First harvest dry matter yield (g/plant)
PR22	Total DM yield (sum of 3 harvests) (g/plant)
PR23	First harvest/total harvest ratio (%)
PR24	Crude protein ratio (%)
PR25	ADF ratio (%)
PR26	NDF ratio (%)
PR27	Seed yield (g/plant)

### Molecular characterization

#### DNA isolation

Young leaf tissues were stored at − 20 °C, genomic DNA isolation was performed using the CTAB method, and the amount and purity were checked spectrophotometrically^24^. All DNA samples were standardized to approximately 50–100 ng/μl for PCR analysis^36^.

#### ISSR analyses and gel imaging

Prior to the main analysis, a total of 22 ISSR primers were initially evaluated, and a subset of representative genotypes was used to screen 11 primers for reproducibility, band clarity, and polymorphism. Based on these criteria, 5 primers were selected for the final analysis (Table 2). PCR amplifications were performed using the Bio-Rad MyCycler device, and the products were separated by electrophoresis in 0.5X TBE buffer on a 2% agarose gel to ensure better resolution of ISSR banding patterns. Gels were stained with ethylenediaminetetraacetic acid and photographed under UV light. Clear and distinct bands were coded as “present” (1) or “absent” (0), while indistinct bands were not included in the evaluation^27^.

**Table 2:** 5’ and 3’ linked ISSR primer sequences used in screening alfalfa populations and varieties in the study.

Primary	Nucleotide sequence (5’3’)	Primary	Nucleotide sequence (5’-3’)
LOL1	(CT)_8_AC	PHV3	GCC(CT)_8_
LOL2	(CT)_8_GC	PHV4	GGC(GT)_8_
LOL3	(CA)_6_AC	PHV5	ACG(CA)_8_
LOL4	(CA)_6_GT	PHV6	CCA(CT)_8_
LOL5	(GA)_6_GG	PHV7	GTG(GT)_8_
LOL6	(GT)_6_GG	PHV8	GAG(CA)_8_
LOL7	(GA)_6_CC	UBC-807	(AG)_8_T
LOL8	(GT)_6_CC	UBC-810	(GA)_8_T
LOL9	(CAC)_3_GC	UBC-811	(GA)_8_C
LOL10	(GAG)_3_GC	UBC-812	(GA)_8_A
LOL11	(CTC)_3_GC	UBC-826	(AC)_8_C

### Statistical analysis

Measurements were conducted on 10 randomly selected plants from each genotype, and each genotype was represented by 40 plants in the field. The experimental evaluation was based on individual plant observations rather than plot-level measurements. Data were collected over two growing seasons, while seed yield (PR27) was recorded only in the second year. Descriptive statistics, including mean, minimum, maximum, standard deviation were calculated using SAS 9.0 statistical software. Genetic similarity among genotypes was determined using the Jaccard similarity coefficient based on ISSR data^37^.

Principal component analysis (PCA), principal coordinate analysis (PCoA), Cluster UPGMA analysis, heatmap, and correlation analyses were performed on the morphological, phenological, yield, and quality parameter data using the R statistical software (version 4.5.1; R Core Team, 2025). The factoextra, pheatmap, corrplot, ComplexHeatmap, and ggplot2 packages were used for the analyses and graphical visualizations, respectively. All analyses were performed with high-resolution visualization and reproducibility principles in mind.

## Results and discussion

In this study, 27 morphological, phenological, yield, and quality parameters (PR1-PR27) were evaluated in 60 local alfalfa (*Medicago sativa* L.) genotypes collected from the Lakes Region. Descriptive statistical results showed a wide variation among genotypes. This variation provides a strong potential base for genetic improvement and represents an important resource for regional breeding programs (Table 3).

**Table 3:** Mean, minimum and maximum values and standard deviation of observed parameters in alfalfa (*n* = 360).

Parameters	Mean	Min	Max	SD
PR1	3.75	1	8	1.72
PR2	6.32	1	10	2.38
PR3	5.01	2	9	1.70
PR4	3.84	1	8	1.43
PR5	3.35	1	7	1.24
PR6	3.83	1	9	1.77
PR7	57.67	14	95	18.86
PR8	70.71	10	100	27.08
PR9	2.88	1	8	1.36
PR10	2.41	1	9	1.88
PR11	55.76	19	86	16.27
PR12	60.46	24	89	16.04
PR13	4.89	1	9	2.21
PR14	3.55	1	6	1.23
PR15	24.27	8	45	8.60
PR16	32.38	12	53	9.33
PR17	4.55	1	7	0.86
PR18	13.69	4.56	21.46	3.13
PR19	249.4	235	264	5.20
PR20	261.78	244	276	5.95
PR21	37.36	13.56	79.77	16.72
PR22	78.77	29.85	175.84	40.39
PR23	0.34	0.30	0.63	0.05
PR24	16.67	11.78	21.86	1.94
PR25	31.91	25.84	39.89	3.01
PR26	42.64	35.85	51.79	2.94
PR27	13.84	6.48	22.56	3.55

### Morphological, phenological, yield, and quality characteristics

#### Seasonal growth characteristics (PR1-PR6)

Winter dormancy (PR1) averages 3.75, indicating that genotypes exhibit moderate dormancy and a balance between winter hardiness and early spring growth in temperate regions^38^. The plant coverage area is high in spring (PR2 = 6.32) and decreases in summer (PR3 = 5.01) and autumn (PR4 = 3.84), reflecting the typical seasonal growth pattern of alfalfa. The low cover rate during winter (PR5 = 3.35) indicates the effect of cold conditions, while the two-week growth rate after mowing (PR6 = 3.83) indicates moderate regrowth potential^39^.

#### Resilience and spread ability (PR7–PR8)

After the second year, plant viability was found to be 57.67% on average, indicating differences in lifespan among genotypes. The spread rate was high (PR8 = 70.71%), indicating strong root crown development and shoot formation ability. This characteristic provides advantages in terms of limiting weed competition and post-harvest cover formation^40,41^.

#### Weed competition and growth form (PR9–PR10)

Weed infestation is low (PR9 = 2.88), indicating that genotypes form a competitive canopy. Growth form (PR10 = 2.41) is generally erect, providing advantages in post-harvest regrowth and mechanical harvesting^42^.

#### Morphological characteristics (PR11–PR17)

Plant height (PR11 = 55.76 cm) and main stem length (PR12 = 60.46 cm) were moderate and showed significant variation among genotypes. This reflects genetic resource diversity for breeding programs^43^. Leaf size (PR13 = 4.89) is an important indicator for plant growth and yield prediction^44^. Root crown diameter (PR14 = 3.55), branch (PR15 = 24.27), and bud number (PR16 = 32.38) were high, indicating regrowth potential under intensive cropping conditions. Root crown depth (PR17 = 4.55) supports drought adaptation with a deep root structure. These traits are critically important for selection to improve forage yield and quality^45^.

#### Phenological characteristics (PR18–PR20)

The harvested area (PR18 = 13.69 cm²) showed moderate differences between genotypes. Flowering initiation (PR19 = 249.4 days) and completion (PR20 = 261.78 days) occurred in the late season. Flowering time is a key phenological characteristic in terms of alfalfa nutrient content and harvest quality^46,47^.

#### Yield and quality characteristics (PR21–PR27)

The first form dry matter yield (PR21 = 37.36 g/plant) is moderate, while the total dry matter yield (PR22 = 78.77 g/plant) is high; the high standard deviation indicates significant variation among genotypes. The ratio of first cut to total yield (PR23 = 0.34) remained constant. Forage yield is a complex trait controlled by genetic and environmental factors^48^. The breeding process is time-consuming due to the perennial structure, inbreeding depression, and long selection cycle^49^. Seed yield (PR27 = 13.84 g) is moderate^50^. The high crude protein (PR24 = 16.67%), moderate ADF (PR25 = 31.91%), and NDF (PR26 = 42.64%) values of alfalfa indicate high feed quality. Breeding programs should aim for a combination of both high yield and high quality^51^.

The wide variation in morphological parameters reflects the ability of the Lakes Region populations to adapt to environmental factors. In particular, traits such as plant height, leaf size, and root crown depth play a critical role in drought tolerance and biomass production. Genetic diversity in these parameters provides a rich source for selection in regional breeding programs^52^.

### PCA-biplot analysis

**Fig. 3:**
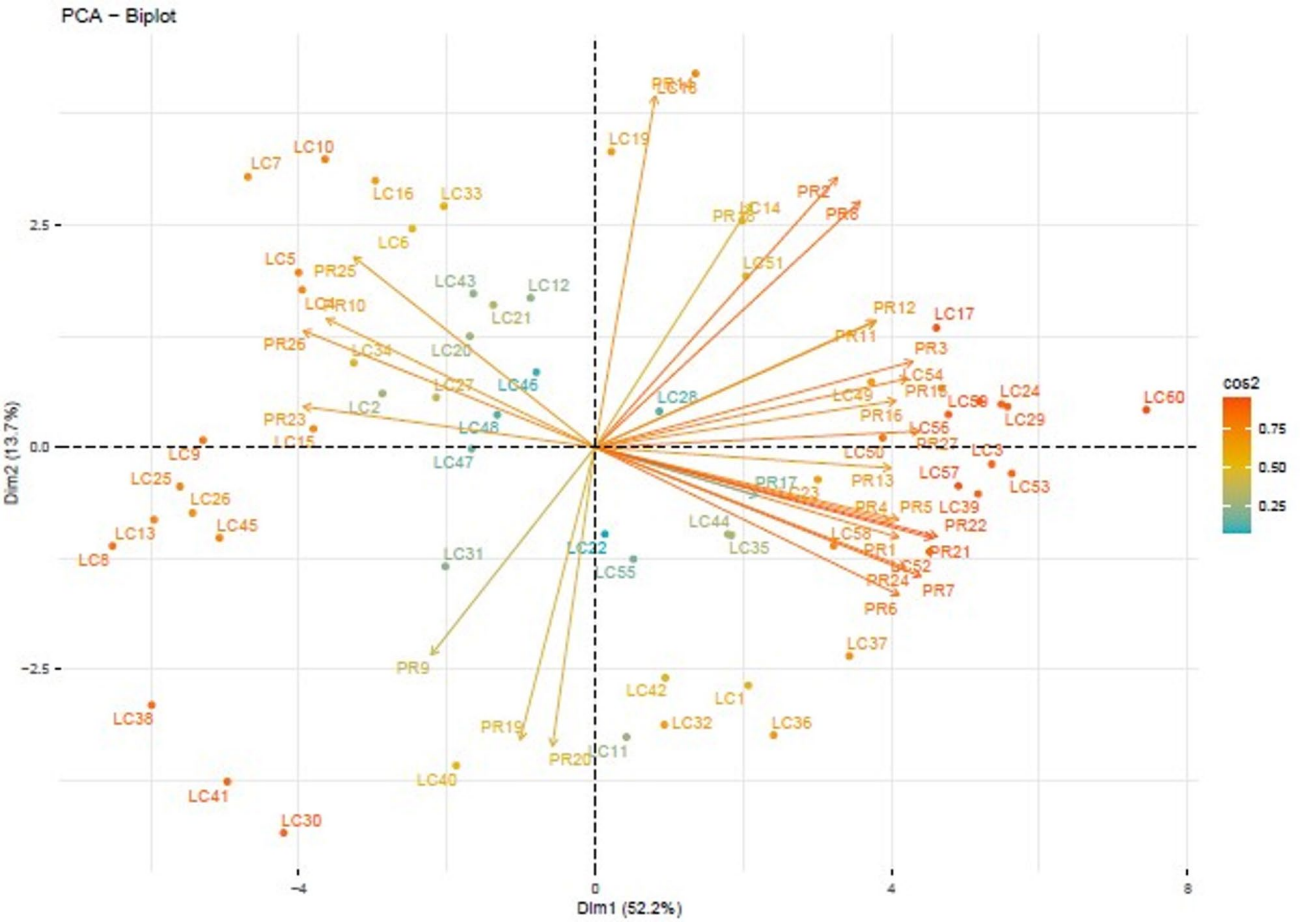
Principal component biplot illustrating the distribution of 60 alfalfa genotypes and 27 phenotypic traits. Vectors represent trait loadings, and colors indicate the cos² contribution of each variable to the principal components.

PCA-biplot analysis revealed clear relationships among agronomic, morphological, and quality traits and helped explain the major sources of variation among genotypes (Fig. 3). The first two principal components (Dim1 and Dim2) accounted for 65.9% of the total variation, indicating a reliable representation of the dataset. Dim1 was primarily associated with yield-related and growth traits (PR7, PR8, PR11, PR12, PR21, PR22), reflecting biomass production capacity. Genotypes located along this axis exhibited higher yield potential and persistence. In contrast, Dim2 was mainly related to quality and phenological traits, with PR24–PR26 contributing strongly, while PR19–PR20 represented variation in developmental timing.

The distribution of genotypes along these axes suggests a clear trade-off between yield and quality components, which is consistent with previous studies in alfalfa and forage crops^53^. Early-maturing genotypes clustered in the upper region of Dim2, whereas late-maturing types were located in the lower region, indicating adaptation to different environmental and management conditions. Overall, these results highlight the importance of multi-trait selection strategies, where combining genotypes from different regions of the PCA space can support the development of varieties with both high yield and improved forage quality.

### Heat map analysis

**Fig. 4:**
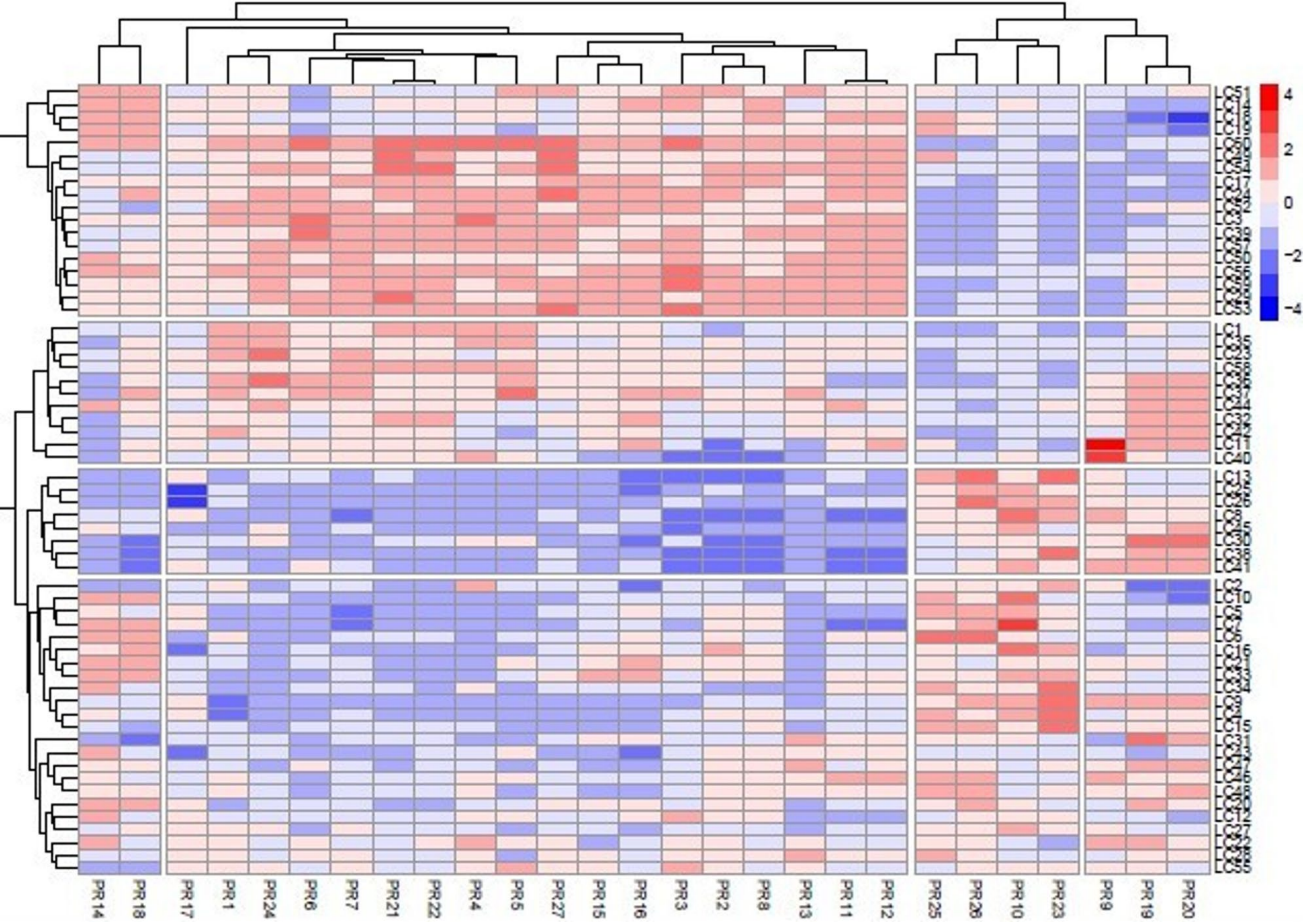
Heat map showing standardized (Z-score) variation among 60 alfalfa genotypes and 27 phenotypic traits. Red indicates higher-than-average trait values, while blue indicates lower-than-average values.

The generated heat map played a significant role in determining the structural relationships between genotypes by simultaneously visualizing the average and variation levels of 27 parameters (Fig. 4). This visualization facilitated the interpretation of multidimensional data by revealing the clustering tendencies of the parameters^54^. The analysis revealed distinct clusters among morphological and yield parameters, and the vertical dendrogram showed that the genotypes formed five main groups based on their genetic similarity. In particular, the clustering of genotypes originating from LC1, LC6, LC40, and LC45 in subclusters suggests that eco-geographic isolation and local adaptation have increased genetic differentiation^55^. In contrast, the distribution of LC13, LC16, and LC46 genotypes across different clusters suggests that gene flow may be higher in these regions^56^. In the horizontal dendrogram, PR6, PR7, and PR8 were grouped in the same subcluster and associated with plant vigor; PR21, PR22, and PR24–PR26 were associated with yield and quality^57^. These findings reveal that the Lake District populations exhibit both high genetic diversity and a pattern of regional adaptation^58^.

### Pearson correlation analysis

**Fig. 5:**
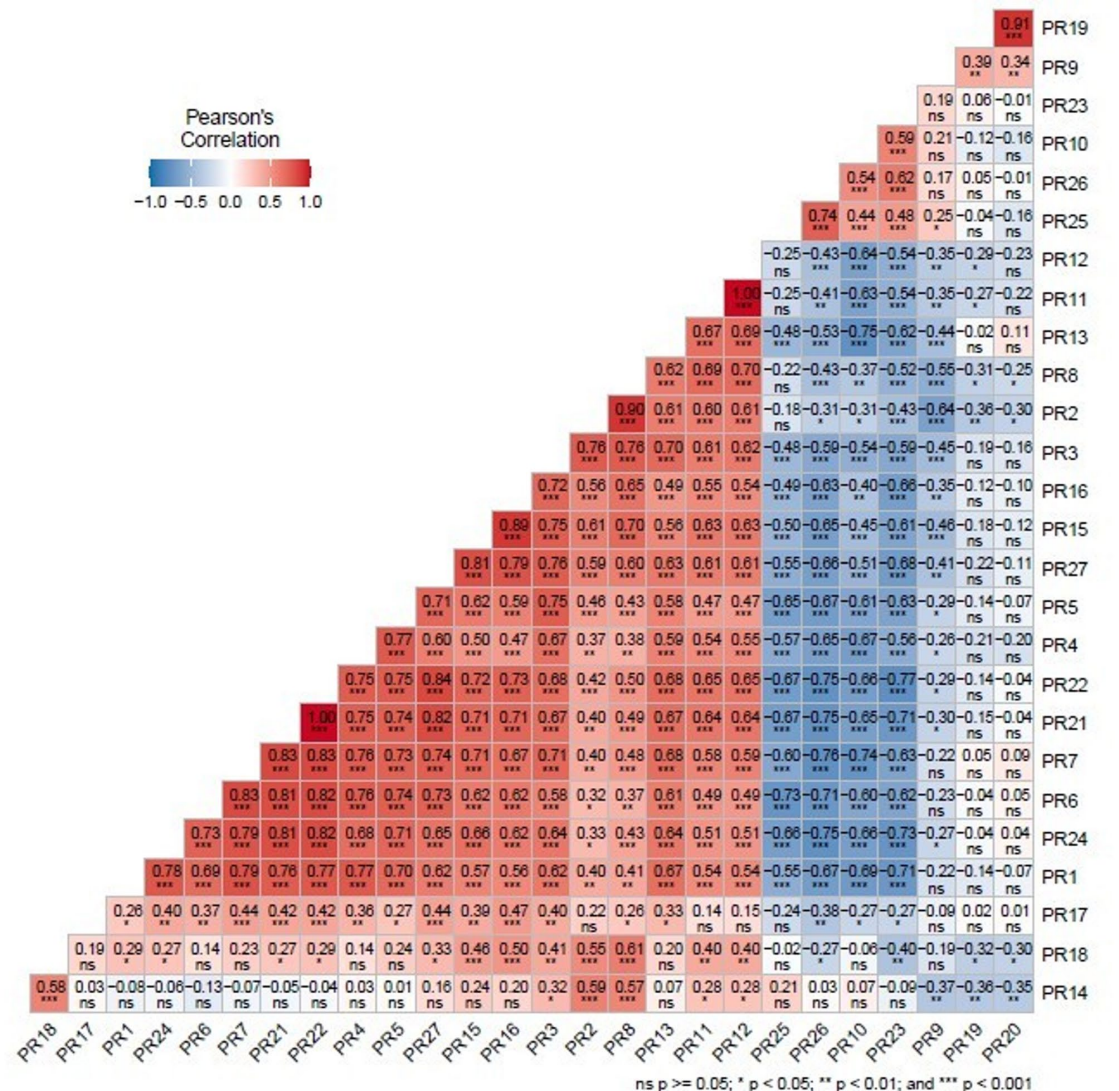
Pearson correlation matrix showing the relationships among 27 (PR1-27) parameters studied in alfalfa populations. The color scale represents the correlation coefficient; red tones indicate positive correlation and blue tones indicate negative correlation. ***, **, and * indicate *p* < 0.001, *p* < 0.01, and *p* < 0.05, respectively.

The correlation matrix was created to evaluate linear relationships between parameters and determine how traits influence each other (Fig. 5). This analysis is particularly important in species such as alfalfa, where multiple breeding criteria are applied^59^. According to the analysis results, strong positive correlations (*r* = 0.71–0.89, *p* < 0.001) were found among the yield components (PR7, PR8, PR11, PR12, PR21, PR22)^60^. These relationships indicate that genotypes with high biomass also exhibit strong root and stem development. Positive correlations were found between quality parameters (PR24–PR26) and negative correlations with yield components; this is consistent with the finding that high yield is often accompanied by a decrease in quality parameters^21^. Positive correlations (*r* = 0.55–0.78) were found among root crown traits (PR14–PR17), while negative correlations (*r* = − 0.41 to − 0.53) were found with phenological traits (PR19–PR20)^61^. These opposite relationships require the creation of balanced selection indices in breeding programs. The high correlation between yield parameters indicates that selection within this group will be effective in increasing yield; however, due to the independent variation of quality parameters, they require a separate breeding strategy (Fig. 5).

### Molecular characterization

**Fig. 6:**
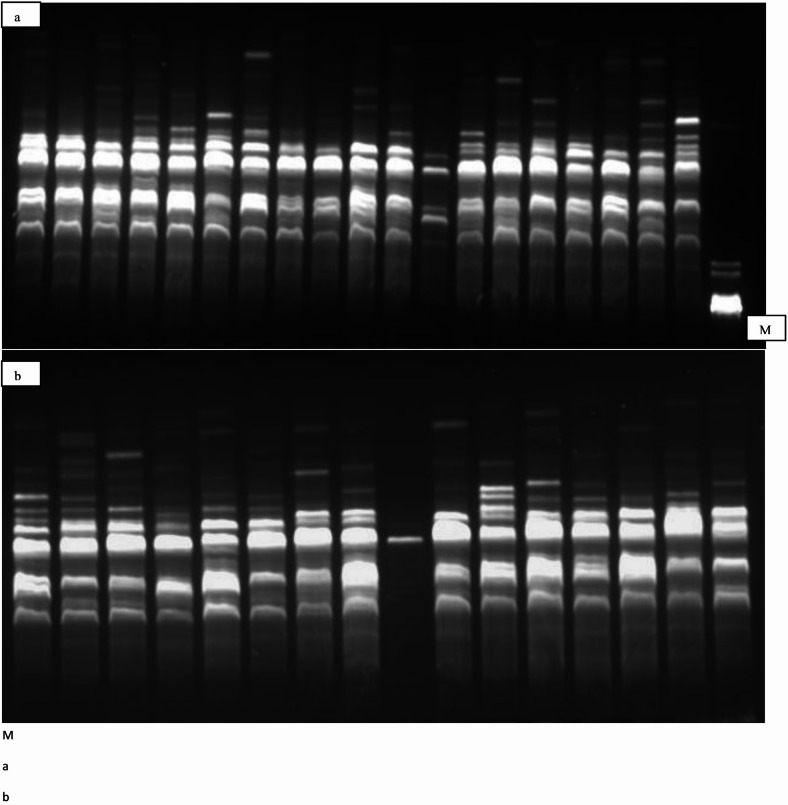
Representative ISSR banding patterns obtained from selected primers. (**a**) Banding profile generated using the LOL4 primer (M = λ/HindIII molecular weight marker). (**b**) PCR amplification profiles obtained using selected ISSR primers (LOL8–10 and PHV5–7) across representative alfalfa genotypes.

This study used ISSR (Inter Simple Sequence Repeat) markers to determine genetic differences among 60 local alfalfa populations and four registered varieties. A total of 22 ISSR primers were initially considered, of which 11 were screened for reproducibility, band clarity, and polymorphism (Fig. 6). Based on these criteria, five primers (LOL1, LOL2, LOL4, LOL8, and PHV3) were selected for further analysis. Bands that were clearly separated on the gels were evaluated as polymorphic and coded in binary format (1/0) to create a data matrix. A total of 71 bands were obtained, 31 of which were polymorphic. On average, there were 14.2 bands per primer, of which 6.2 were polymorphic, giving a polymorphism rate of 41.3%. The highest polymorphism rate was observed with the LOL2 primer (61.1%). ISSR profiles indicate a high level of genetic diversity among the populations studied, leading to the conclusion that this variation could constitute a valuable genetic resource for the development of new alfalfa varieties. Similarly, previous studies have also reported that ISSR markers are an effective tool for revealing genetic variation in alfalfa^57,58^.

### PCoA-biplot analysis

**Fig. 7:**
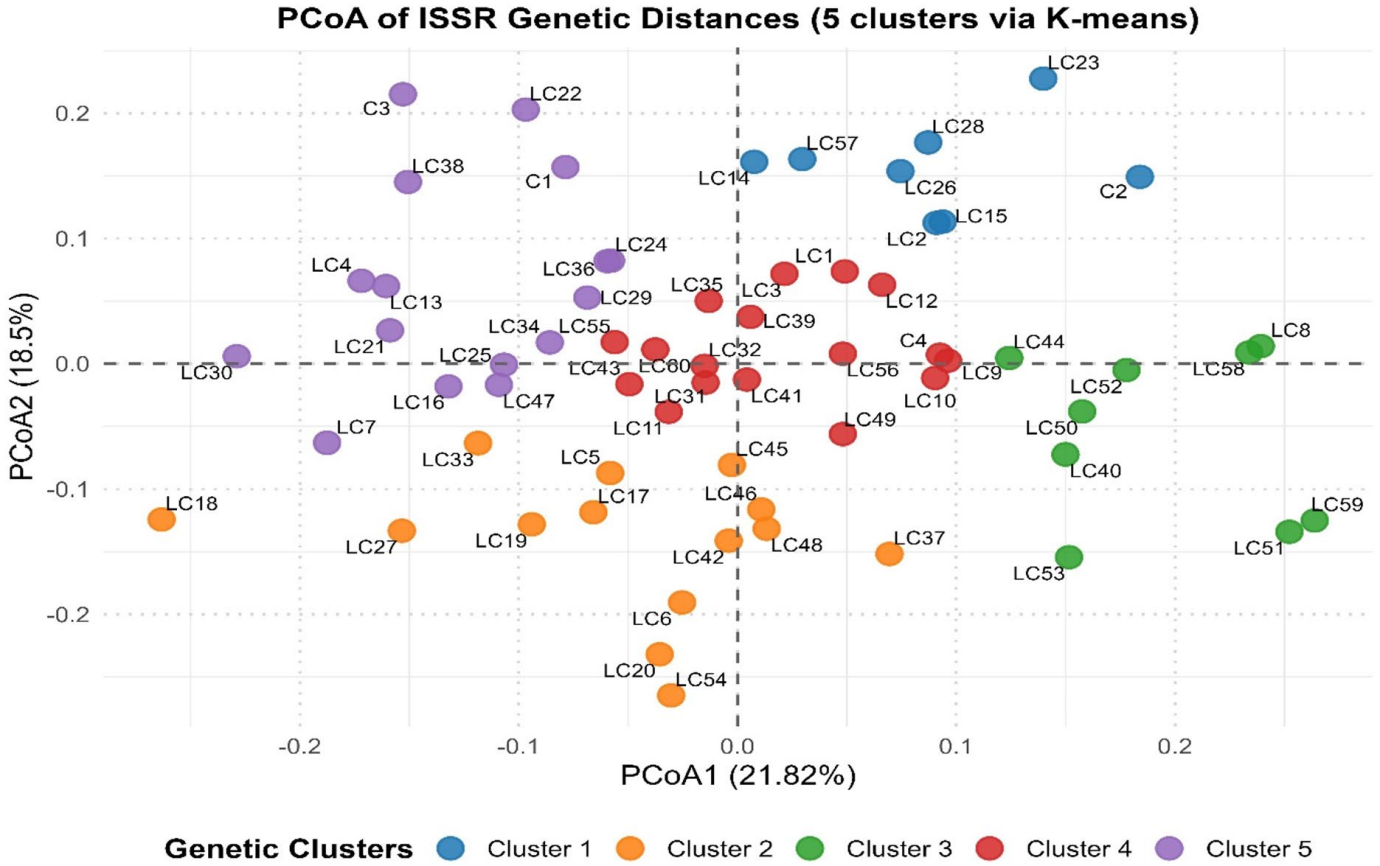
Principal Coordinate Analysis (PCoA) of 60 local and 4 control alfalfa (Medicago sativa L.) genotypes based on ISSR-derived genetic distance matrix. The first two coordinates (PCoA1 = 21.82%, PCoA2 = 18.50%) jointly explain 50.32% of total genetic variation. Each color represents one of the five genetic clusters identified by the K-means algorithm (Cluster 1–5). Genotypes grouped in the same color belong to genetically similar groups, while those in different colors indicate distinct genetic backgrounds.

Principal coordinate analysis (PCoA) has been widely used to represent genetic diversity in breeding programs for different plant species. In all these studies, relationships between genotypes were primarily revealed by coordinate analysis^62^. PCoA analysis based on ISSR markers revealed distinct genetic differentiation among 60 local alfalfa (*Medicago sativa* L.) populations and 4 control varieties (Fig. 7). The first two axes explain 40.32% of the total variation (Dim1 = 21.82%, Dim2 = 18.50%). This ratio represents a high level of differentiation reflecting the multidimensional structure of genetic diversity in natural populations. The five genetic clusters defined by K-means classification are clearly separated by color in the graph: Cluster 1 is located near the center of the graph, representing average genetic variation. Cluster 2 and Cluster 3 are clustered in the positive direction of the PCoA1 axis and contain genotypes rich in alleles (e.g., LC54, LC59, LC53, LC6, LC45) with high genetic distances. Cluster 4 contains culture-derived genotypes such as LC23 and LC28 with C1–C3 control varieties, and the location of this group in the lower region indicates genetic narrowing due to breeding history. Cluster 5, located in the upper left, reflects a differentiated structure due to local adaptation or geographical isolation, with genotypes LC18, LC30, LC7, LC21, and LC47. These results are consistent with the view that this may be related to alfalfa being an allogamous and heterozygous species^63^. Overall, ISSR markers strongly reflected the population structure, revealing high genetic diversity and structural differentiation. These findings indicate that local LC genotypes offer significant potential as a resource for conservation and evaluation in breeding programs. Molecular-level variation is an important resource for breeding programs^64,65^.

### Cluster analysis

**Fig. 8:**
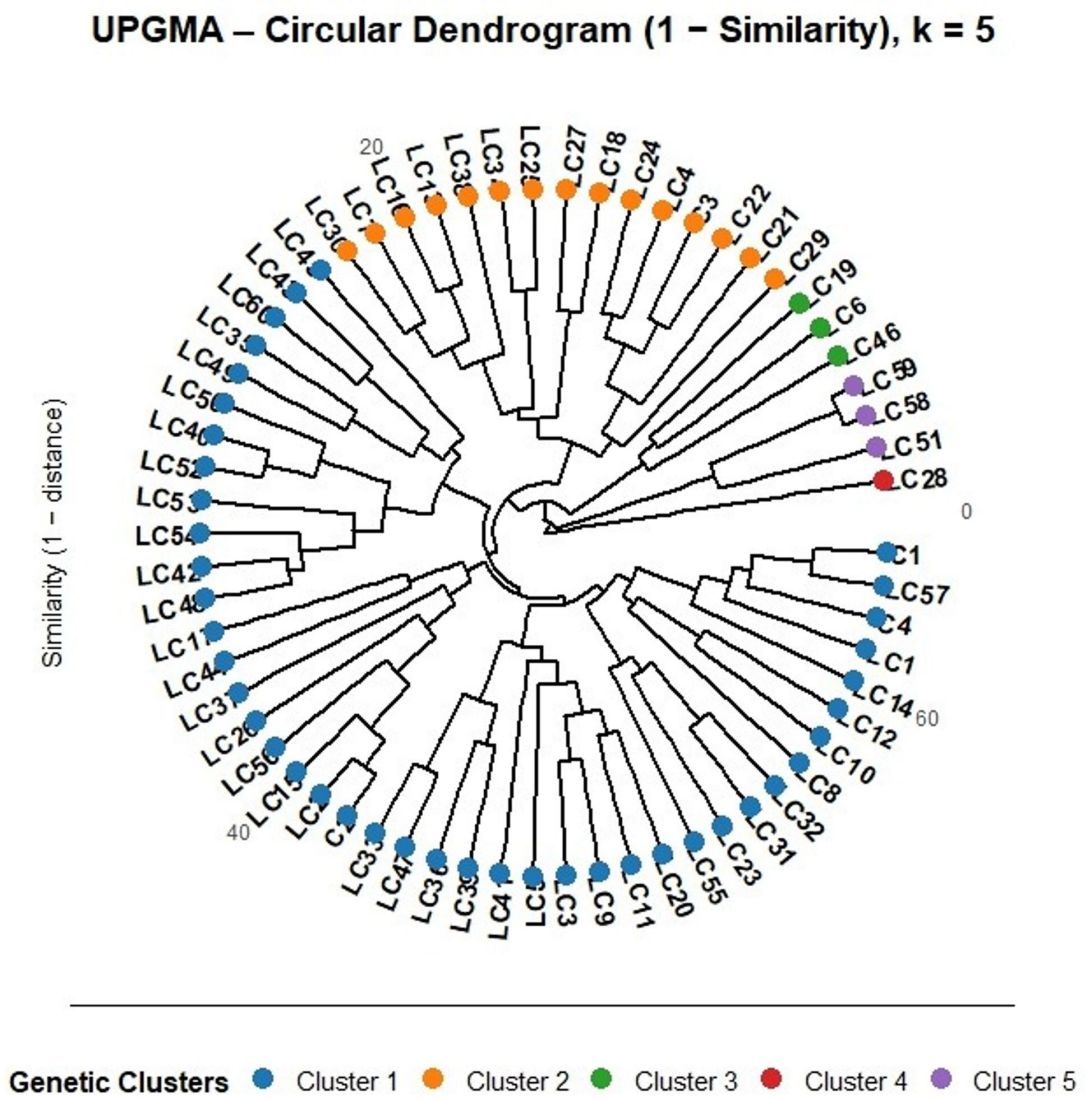
Circular UPGMA dendrogram showing the hierarchical genetic relationships of 60 landrace and 4 control alfalfa (Medicago sativa L.) genotypes based on ISSR similarity coefficients. Five main genetic clusters (Clusters 1–5) are colored (Blue, Orange, Green, Red, Purple) consistent with PCoA analysis. Genotypes located on the same colored branch share higher genetic similarity, while greater branch distances indicate higher genetic divergence.

The circular UPGMA dendrogram revealed a distinct hierarchical genetic structure among the 64 genotypes examined (60 local populations + 4 control varieties) (Fig. 8). Five major genetic clusters were identified, each showing high internal homogeneity and clear inter-cluster differences. Cluster 1, predominantly containing local genotypes, reflects a common genetic basis resulting from adaptation to similar environmental conditions in the Lakes Region. Cluster 2 and Cluster 3 encompass genotypes with moderate genetic distance, while Cluster 4 and Cluster 5 are more distantly located and contain genetically distinct genotypes (e.g., LC51, LC58, LC28). This clustering pattern is highly consistent with PCoA results and demonstrates that ISSR markers strongly reflect population differentiation.

The branching pattern reveals that the populations have a multi-ancestral genetic structure, meaning that the alfalfa genotypes in the region may have evolved from more than one ancestor. The clustering analysis results of Touil et al.^26^ show that the different clusters obtained include both local alfalfa populations and cultivated alfalfa populations, indicating that population clustering is not related to geographic origin. This observation is partially consistent with the results drawn from the clustering analysis in this study. This difference may be attributed to the relatively limited geographical origin of the populations under study, which could lead to stronger local adaptation and more structured genetic patterns. However, Shen et al.^66^ noted that alfalfa varieties in North America and South America clustered into distinct groups, consistent with the migration and cultivation history of alfalfa. Our research findings emphasize that the Lakes Region populations carry rich genetic variation and represent a valuable genetic resource for breeding programs.

Population structure analysis allows us to understand the genetic diversity within a given collection and identify the appropriate population for relationship mapping. Cluster analysis demonstrated the ability and usefulness of ISSR markers in examining differentiation and relatedness among alfalfa populations. UPGMA-based cluster analysis and PCoA results showed that alfalfa genotypes from the Lakes Region of Türkiye were divided into different groups that were significantly related based on their geographical origins.

### Heat map analysis

**Fig. 9:**
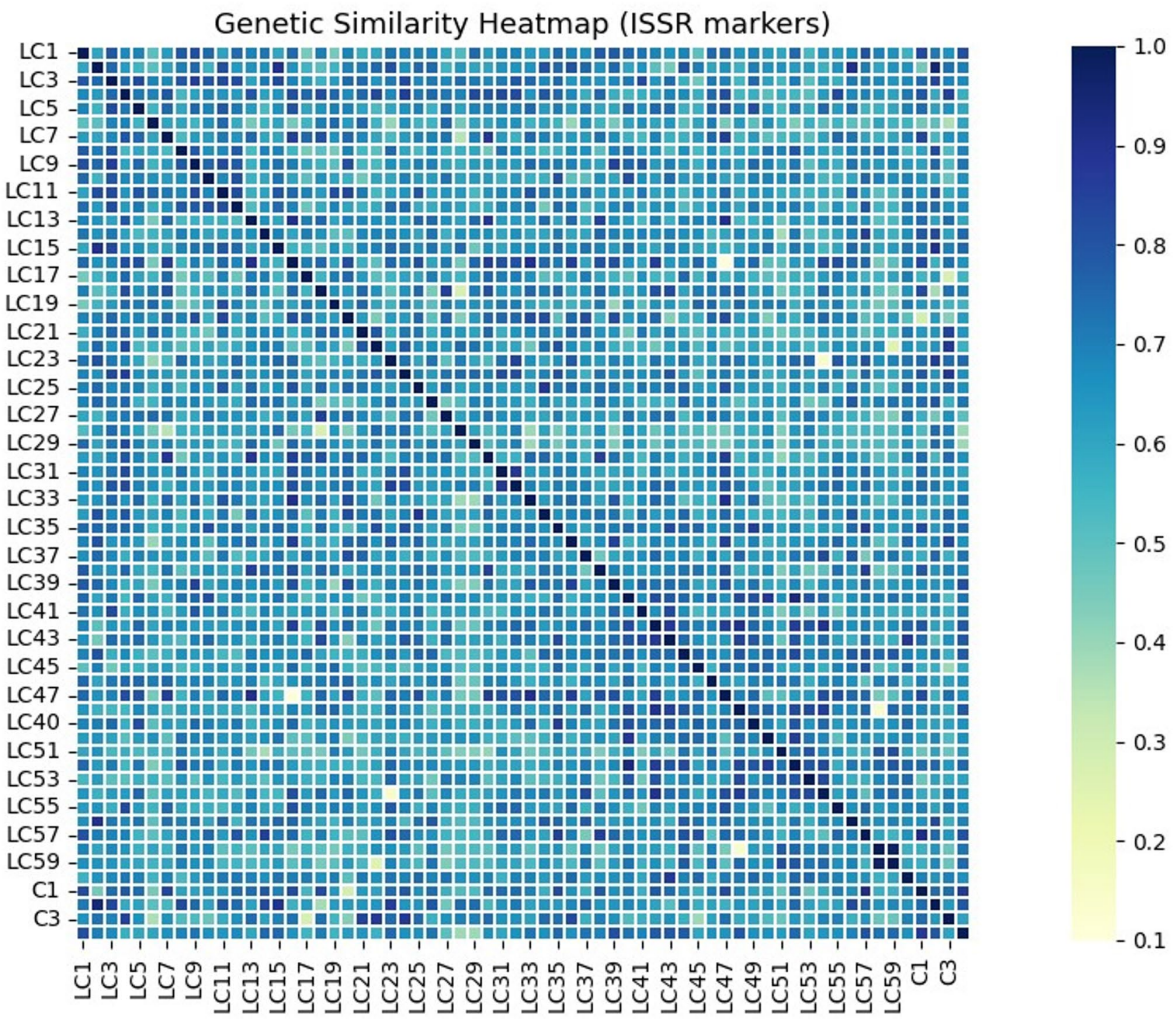
ISSR-based Genetic Similarity Heatmap (1 − Distance) for 60 alfalfa genotypes and 4 cultivars. Darker colors represent higher similarity coefficients, while lighter shades indicate lower genetic similarity. Hierarchical clustering (UPGMA, average linkage) shows the genetic relationships among genotypes.

The genetic similarity heat map based on ISSR markers showed the pairwise genetic relationships among 60 LC genotypes and 4 control varieties through color intensity (Fig. 9). Dark blue tones on the color scale represent high genetic similarity, while light green–yellow tones represent low genetic similarity. Dark colorations near the diagonal axis indicate that genotypes within their own clusters share high genetic similarity and confirm the clustering patterns identified in K-means and UPGMA analyses. The distinct color blocks on the map clearly show that the population is organized around five main genetic groups. The control varieties (C1–C4) are located in lighter tones compared to the LC genotypes, indicating that they have different genetic origins and represent the overall variation range of the population. In conclusion, the heat map based on ISSR markers accurately reflected intrapopulation variation and genetic differentiation between clusters, visually supporting the comprehensive structure of the K-means and UPGMA analyses. Cluster analysis is used as it is an effective tool for determining the degree of genetic diversity among tested genotypes based on their performance and contributing traits^67^. Furthermore, multivariate statistical methods such as Principal Component Analysis (PCA) and Principal Coordinate Analysis (PCoA)^68^ have successfully supported researchers in studying variability in germplasm collections of many species, as well as in numerical classifications.

## Conclusion

Analyses of morphological, phenological, yield, and quality characteristics, together with molecular data, revealed that the 60 local populations (LC), represented by clonally propagated genotypes, along with 4 control varieties (C), exhibit a wide range of genetic variation. Both datasets indicate a high level of differentiation among populations and that morphological variation is largely based on genetic factors. Morphological and phenological analyses revealed that the genotypes LC3, LC17, LC24, LC29, LC39, LC49, LC53, LC54, LC57, and LC60 possess superior traits such as early flowering, rapid post-emergence growth, tall plant height, large leaf area, and strong root crown. These genotypes were found to have high dry forage and seed yield, high crude protein content, and low ADF–NDF values. Superior genotypes were identified based on a combined evaluation of yield, quality, morphological traits and molecular clustering rather than on PCA results alone. At the molecular level, PCoA results based on ISSR markers showed significant genetic differentiation between populations. The first two axes explained 40.32% of the total variation, and K-means analysis divided the genotypes into five main groups. The LC24, LC49, LC54, LC57, and LC60 genotypes were located in genetically distinct clusters and were genetically distant from some control varieties. The combined evaluation of morphological and molecular data revealed that the variation was largely genetically based and that ISSR markers accurately reflected the population structure. It was concluded that alfalfa genotypes possess complementary characteristics in terms of morphological performance, forage quality, and molecular diversity. Therefore, the genotypes LC3, LC17, LC24, LC29, LC39, LC49, LC53, LC54, LC57, and LC60 are recommended as the most suitable candidate materials for use in synthetic variety breeding programs to develop a new alfalfa variety, due to their high yield and quality potential and their representation of different genetic clusters. This multifaceted evaluation, supported by ISSR data, is of strategic importance for the conservation of Türkiye’s local alfalfa populations and their effective use in breeding programs.

A new synthetic alfalfa variety called “Albatur” was developed using the genotypes selected in this study and was officially registered by the Turkish Ministry of Agriculture and Forestry in 2023. The method and selection criteria used in this study can be a valuable resource for those working in alfalfa breeding.

## Supplementary Information

Below is the link to the electronic supplementary material.

Supplementary Material 1

## Data Availability

Data will be made available on request. Contacts: sebahattinalbayrak@omu.edu.tr (Sebahattin ALBAYRAK).

## References

[1] Liu, S., Wang, K. & Wang, J. Effects of alfalfa and oatgrass on soil nutrients in newly cultivated land on the Loess Plateau, China. *Sci. Rep.* **15**, 35888. 10.1038/s41598-025-19895-7 (2025). 41087721 10.1038/s41598-025-19895-7 PMC12521648

[2] Feng, Y. et al. Yield and quality properties of alfalfa (Medicago sativa L.) and their influencing factors in China. *Eur. J. Agron.* **141**, 126637. 10.1016/j.eja.2022.126637 (2022).

[3] Albayrak, S., Oten, M., Turk, M. & Alagoz, M. An investigation on improved source population for the alfalfa (Medicago sativa L.) breeding. *Legume Res.* **41**, 828–832. 10.18805/LR-420 (2018).

[4] Arshad, M., Feyissa, B. A., Amyot, L., Aung, B. & Hannoufa, A. MicroRNA156 improves drought stress tolerance in alfalfa (Medicago sativa L.) by silencing SPL13. *Plant. Sci.* **258**, 122–136. 10.1016/j.plantsci.2017.01.018 (2017). 28330556 10.1016/j.plantsci.2017.01.018

[5] Tian, Z. et al. Effect of genotype and environment on agronomical characters of alfalfa (Medicago sativa L.) in a typical acidic soil environment in southwest China. *Front. Sustain. Food Syst.* **7**, 1144061. 10.3389/fsufs.2023.1144061 (2023).

[6] Greveniotis, V. et al. Modeling stability of alfalfa yield and main quality traits. *Agriculture* **14**, 542. 10.3390/agriculture14040542 (2024).

[7] Rojas-Garcia, A. et al. Yield components of alfalfa (Medicago sativa L.) varieties. *Agrociencia* **51**, 697–708 (2017).

[8] Avci, M., Hatipoglu, R., Çinar, S. & Kiliçalp, N. Assessment of yield and quality characteristics of alfalfa (Medicago sativa L.) cultivars with different fall dormancy rating. *Legume Res.* **41**, 369–373. 10.18805/LR-364 (2018).

[9] Eckberg, J. O., Wells, S. S., Jungers, J. M., Lamb, J. F. S. & Sheaffer, C. C. Alfalfa forage yield, milk yield, and nutritive value under intensive cutting. *Agrosyst Geosci. Environ.* **5**, e20246. 10.1002/agg2.20246 (2022).

[10] Alzahrani, O. R. et al. Evaluation of genetic diversity among Saudi Arabian and Egyptian cultivars of alfalfa (Medicago sativa L.) using ISSR and SCoT markers. *J. Taibah Univ. Sci.* **17**, 2194187. 10.1080/16583655.2023.2194187 (2023).

[11] Liatukiene, A., Norkevičienė, E., Danytė, V. & Liatukas, Ž. Diversity of agro-biological traits and development of diseases in alfalfa cultivars during contrasting vegetation seasons. *Sustainability* **15**, 1445. 10.3390/su15021445 (2023).

[12] Öten, M. & Albayrak, S. Yield and quality characteristics of alfalfa (Medicago sativa L.) genotypes in the high dormancy group. *J. Agr Sci.* **36**, 293–300. (2021). 10.7161/omuanajas.874970

[13] Wang, S. et al. Dynamics of spring regrowth and comparative production performance of 50 autumn-sown alfalfa cultivars in the coastal saline soil of North China. *Life* **11**, 1436. 10.3390/life11121436 (2021). 34947967 10.3390/life11121436 PMC8706483

[14] Teixeira, E. I., Moot, D. J., Brown, H. E. & Fletcher, A. L. The dynamics of lucerne (Medicago sativa L.) yield components in response to defoliation frequency. *Eur. J. Agron.* **26**, 394–400. 10.1016/j.eja.2006.12.005 (2007).

[15] Sayed, M. R. I. et al. Genetic diversity, analysis of some agro-morphological and quality traits and utilization of plant resources of alfalfa. *Genes* **13**, 1521. 10.3390/genes13091521 (2022). 36140688 10.3390/genes13091521 PMC9498742

[16] He, Y. et al. Adaptability evaluation of four alfalfa (Medicago sativa L.) varieties in cold regions of northern China: Based on multidimensional indicators, metabolites, and soil microbial community analysis. *Ind. Crops Prod.* **234**, 121657. 10.1016/j.indcrop.2025.121657 (2025).

[17] Okumuş, O. et al. Integrating machine learning and in vitro screening to evaluate drought and temperature stress responses for Vicia species. *Processes* **13** (6), 1845. 10.3390/pr13061845 (2025).

[18] Escobar-Gutiérrez, A. J. & Ahmet, L. Q. Early detection of phenotypic diversity of alfalfa (Medicago sativa L.) in response to temperature. *Plants* **12** (18), 3224. 10.3390/plants12183224 (2023). 37765388 10.3390/plants12183224 PMC10536524

[19] Jungers, J., Cherney, J., Martinson, K., Jaqueth, A. & Sheaffer, C. Forage nutritive value of modern alfalfa cultivars. *Crop Forage Turfgrass Manag*. **6**, 20076. 10.1002/cft2.20076 (2020).

[20] Fustini, M. et al. Effect of undigested neutral detergent fiber content of alfalfa hay on lactating dairy cows: Feeding behavior, fiber digestibility, and lactation performance. *J. Dairy. Sci.*10.3168/jds.2016-12266 (2017). 28342598 10.3168/jds.2016-12266

[21] Tucak, M., Horvat, D., Čupić, T., Krizmanić, G. & Ravlić, M. Assessment of alfalfa populations for forage productivity and seed yield potential under a multi-year field trial. *Agronomy* **13**, 349. 10.3390/agronomy13020349 (2023).

[22] Biswas, A. et al. Phenomics-assisted selection for herbage accumulation in alfalfa (Medicago sativa L.). Front. *Plant. Sci.* **12**, 756768. 10.3389/fpls.2021.756768 (2021). 10.3389/fpls.2021.756768 PMC8689394 34950163

[23] Zhao, J., Huang, R., Yang, K., Ma, C. & Zhang, Q. Effects of nitrogen and phosphorus fertilization on photosynthetic properties of leaves and agronomic characters of alfalfa over three consecutive years. *Agriculture* **12**, 1187. 10.3390/agriculture12081187 (2022).

[24] Shi, R., Yang, J., Zhang, Y., Yang, Y. & Shi, F. Selection of alfalfa (Medicago sativa L.) hybrid parents and heterosis analysis of F1 hybrids. *Turk. J. Field Crops*. **27** (2), 235–241. 10.17557/tjfc.1126296 (2022).

[25] Heiba, S. A. A., Ali, R. T., Abdel-Rahman, H. M. & Rashad, S. E. Detected molecular markers for alfalfa (Medicago sativa) using ISSR and SSR under Egyptian conditions. *Int. J. Latest Technol. Eng. Manag Appl. Sci.* **11**, 11 (2022).

[26] Touil, L., Bao, A., Wang, S. & Ferchichi, A. Genetic diversity of Tunisian and Chinese alfalfa (Medicago sativa L.) revealed by RAPD and ISSR markers. *Am. J. Plant. Sci.* **7**, 967–979. 10.4236/ajps.2016.76092 (2016).

[27] Rongxi, S., Lin, F. R., Ping, H. & Zheng, Y. Q. Moderate genetic diversity and genetic differentiation in the relict tree Liquidambar formosana Hance revealed by genic simple sequence repeat markers. *Front. Plant. Sci.* **7**, 1411. 10.3389/fpls.2016.01411 (2016). 27708661 10.3389/fpls.2016.01411 PMC5030344

[28] Rankin, M. Alfalfa faces challenges, but all isn’t lost. Hay & Forage Grower. (2022). https://hayandforage.com/article-4215-alfalfa-faces-challenges-but-all-isn-t-lost.html

[29] Medina, C. A. et al. Pre-breeding in alfalfa germplasm develops highly differentiated populations, as revealed by genome-wide microhaplotype markers. *Sci. Rep.* **15**, 1253. 10.1038/s41598-024-84262-x (2025). 39779777 10.1038/s41598-024-84262-x PMC11711157

[30] El-Ramady, H. et al. Alfalfa growth under changing environments: An overview. *Environ. Biodiv Soil. Secur.* **4**, 201–224 (2020).

[31] Fasoulas, A. C. A moving block evaluation technique for improving the efficiency of pedigree selection. *Euphytica* **36**, 473–478 (1987).

[32] Öten, M. & Albayrak, S. Collection and characterization of alfalfa (Medicago sativa L.) in West Mediterranean coastal zone. *Derim* **31** (2), 81–90. 10.16882/derim.2014.50249 (2014).

[33] Çelik, H. & Kalaycı Karasakal, Ö. Effects of different media, cutting time and IBA doses on rooting of Rockspray Cotoneaster (Cotoneaster horizontalis Decne.) cuttings. *Acad. Agric. J.* **14** (1), 23–38. 10.29278/azd.1616448 (2025).

[34] Prosperi, J. M., Jenczewski, E., Angevain, M. & Ronfort, J. Morphological and agronomic diversity of wild genetic resources of Medicago sativa L. collected in Spain. *Genet. Resour. Crop Evol.* **53**, 843–856. 10.1007/s10722-004-6476-3 (2006).

[35] Albayrak, S. & Türk, M. Changes in the forage yield and quality of legume-grass mixtures throughout the vegetation period. *Turk. J. Agric. For.*10.3906/tar-1202-73 (2013).

[36] Doyle, J. & Doyle, J. Isolation of plant DNA from fresh tissue. *Focus* **12**, 13–15 (1990).

[37] Rohlf, F. J. *NTSYS-pc: Numerical taxonomy and multivariate analysis system, version 2.2* (Exeter Software, 2000).

[38] Wang, C. et al. Yields of alfalfa varieties with different fall-dormancy levels in a temperate environment. *Agron. J.* **101** (5), 1146–1152. 10.2134/agronj2009.0026 (2009).

[39] Castonguay, Y., Laberge, S., Brummer, C. & Volenec, J. J. Alfalfa winter hardiness: A research retrospective and integrated perspective. *Adv. Agron.* **90**, 203–265. 10.1016/S0065-2113(06)90006-6 (2006).

[40] Pan, X. et al. Exploring root system architecture and anatomical variability in alfalfa (Medicago sativa L.) seedlings. *BMC Plant. Biol.* **23**, 449. 10.1186/s12870-023-04469-4 (2023). 37743492 10.1186/s12870-023-04469-4 PMC10519072

[41] Jing, T., Liang, S., Liu, C., Liu, S. & Sun, L. Study on the effects of planting alfalfa (Medicago sativa L.) and adding biochar on soil fertility in jujube orchards. *Agronomy* **15**, 1462. 10.3390/agronomy15061462 (2025).

[42] Jing, F. et al. Analysis of phenotypic and physiological characteristics of plant height difference in alfalfa. *Agronomy* **13**, 1744. 10.3390/agronomy13071744 (2023).

[43] Riasat, S. et al. Genetic diversity estimation in alfalfa (Medicago sativa L.) genotypes using SSR markers, morphological and yield related traits. *J. Agric. Res.* **59** (2), 133–139 (2021).

[44] Moghaddam, A. & Mofidian, S. M. A. Comparison of forage yield and leaf area index of some local and foreign alfalfa (Medicago sativa L.) genotypes. Iran. J. Rangel. Forests Plant Breed. *Genet. Res.* **29** (2), 317–329. 10.22092/ijrfpbgr.2022.356780.1398 (2022).

[45] Awad, I. M. H. Selection response in Berseem (Trifolium alexandrinum L.) and alfalfa (Medicago sativa L.). Ph.D. Thesis, Faculty of Agric., Assiut Univ., Egypt. (2001).

[46] Albayrak, S. & Öten, M. Determination of yield and quality characteristics with general combination abilities of alfalfa (Medicago sativa L.) genotypes in progeny control plots. *Anadolu J. Agr Sci.* **35**10.7161/omuanajas.743199 (2018).

[47] Wang, S. Y. et al. Alfalfa quality improvement and loss reduction technology advances. *Front. Anim. Sci.* **6**, 1550492. 10.3389/fanim.2025.1550492 (2025).

[48] Ren, L., Bennett, J. A., Coulman, B., Liu, J. & Biligetu, B. Forage yield trend of alfalfa cultivars in the Canadian prairies and its relation to environmental factors and harvest management. *Grass Forage Sci.* **76**, 390–399. 10.1111/gfs.12513 (2021).

[49] Annicchiarico, P. & Pecetti, L. Comparison among nine alfalfa breeding schemes based on actual biomass yield gains. *Crop Sci.* **61**, 2355–2370. 10.1002/csc2.20464 (2021).

[50] Ualiyeva, G. T., Sagalbekov, U. M., Baidalin, M. E. & Yancheva, H. Development of a model, selection and evaluation of the source material for the plant breeding of alfalfa varieties with increased seed productivity. *Online J. Biol. Sci.* **22**, 139–148. 10.3844/ojbsci.2022.139.148 (2022).

[51] Tucak, M. et al. Agro-morphological and forage quality traits of selected alfalfa populations and their application in breeding. *Turk. J. Field Crops*. **19** (1), 79–83 (2014).

[52] Acharya, J. P. et al. Breeding alfalfa (Medicago sativa L.) adapted to subtropical agroecosystems. *Agronomy* **10**, 742. 10.3390/agronomy10050742 (2020).

[53] Jing, F. et al. The physiological basis of alfalfa plant height establishment. *Plants* **13**, 679. 10.3390/plants13050679 (2024). 38475525 10.3390/plants13050679 PMC10934537

[54] Hossain, M. M., Kordrostami, M., Goto, F. & Rahimi, M. Spermine treatment improves salinity tolerance in Plantago major by altering growth parameters, biochemical profiles and gene expression. *Sci. Rep.* **15**, 25762. 10.1038/s41598-025-11903-0 (2025). 40670597 10.1038/s41598-025-11903-0 PMC12267852

[55] Liu, H. et al. Geographic isolation and environmental heterogeneity contribute to genetic differentiation in Cephalotaxus oliveri. Ecol. Evol. 13(3), e9869. 10.1002/ece3.9869 (2023). 36919017 10.1002/ece3.9869 PMC10008294

[56] Kesoju, S. R. et al. Gene flow in commercial alfalfa (Medicago sativa subsp. sativa L.) seed production fields: Distance is the primary but not the sole influence on adventitious presence. *PLoS ONE*. **16** (3), e0248746. 10.1371/journal.pone.0248746 (2021). 33765070 10.1371/journal.pone.0248746 PMC7993763

[57] Petolescu, C. et al. Assessment of genetic diversity in alfalfa using DNA polymorphism analysis and statistical tools. *Plants* **13**, 2853. 10.3390/plants13202853 (2024). 39458800 10.3390/plants13202853 PMC11511019

[58] Wang, H., Coulman, B., Bai, Y., Tar’an, B. & Biligetu, B. Genetic diversity and local adaptation of alfalfa populations (Medicago sativa L.) under long-term grazing. *Sci. Rep.* **13**, 1632. 10.1038/s41598-023-28521-3 (2023). 36717619 10.1038/s41598-023-28521-3 PMC9886962

[59] Cao, Z. et al. PCA-driven multivariate trait integration in alfalfa breeding: A selection model for high-yield and stable progenies. *Plants* **14**, 2906. 10.3390/plants14182906 (2025). 41012057 10.3390/plants14182906 PMC12473214

[60] Kebede, G. et al. Biomass yield potential and herbage quality of alfalfa (Medicago sativa L.) genotypes in the central highlands of Ethiopia. *Int. J. Res. Stud. Agric. Sci.* **3** (1), 14–26. 10.20431/2454-6224.0301003 (2017).

[61] Bucciarelli, B. et al. Phenotyping seedlings for selection of root system architecture in alfalfa (Medicago sativa L). *Plant. Methods*. **17**, 125. 10.1186/s13007-021-00825-3 (2021). 34876178 10.1186/s13007-021-00825-3 PMC8650460

[62] Santanna, I. C. et al. Comparison of projection of distance techniques for genetic diversity studies. *Genet. Plant. Breed.*10.4025/actasciagron.v42i1.42483 (2018).

[63] Rameshknia, Y., Rashidi, V., Monirifar, H. & Sabbaghtazeh, E. Genetic diversity analysis of tetraploid alfalfa (Medicago sativa subsp. sativa L.) populations collected from north-west regions of Iran using simple sequence repeat markers. *Genet. Resour. Crop Evol.* **71**, 3603–3612. 10.1007/s10722-024-01858-y (2024).

[64] Rhouma, H. B., Taski-Ajdukovic, K., Zitouna, N., Sdouga, D. & Milic, D. Trifi-Farah, N. Assessment of the genetic variation in alfalfa genotypes using SRAP markers for breeding purposes. *Chil. J. Agric. Res.* **75** (4), 465–471 (2015).

[65] Humphries, A. W. et al. Characterization and pre-breeding of diverse alfalfa wild relatives originating from drought-stressed environments. *Crop Sci.* **61**, 69–88. 10.1002/csc2.20274 (2021).

[66] Shen, C. et al. The chromosome-level genome sequence of the autotetraploid alfalfa and resequencing of core germplasms provide genomic resources for alfalfa research. *Mol. Plant.* **13**, 1250–1261. 10.1016/j.molp.2020.07.003 (2020). 32673760 10.1016/j.molp.2020.07.003

[67] Qiang, H. et al. Molecular diversity and population structure of a worldwide collection of cultivated tetraploid alfalfa (Medicago sativa subsp. sativa L.) germplasm as revealed by microsatellite markers. *PLoS ONE*. **10**, e0124592. 10.1371/journal.pone.0124592 (2015). 25901573 10.1371/journal.pone.0124592 PMC4406709

[68] Hdira, S. et al. Morpho-physiological, biochemical and genetic esponses to salinity in Medicago truncatula. *Plants* **10**, 808. 10.3390/plants10040808 (2021). 33924007 10.3390/plants10040808 PMC8072551

